# Evaluating the Oxidative Balance Score for Peripheral Artery Disease Risk: Integrating Epidemiologic Modeling and SHAP‐Interpretable Machine Learning in NHANES

**DOI:** 10.1002/fsn3.70798

**Published:** 2025-08-17

**Authors:** Zeyi Zhou, Han Lee, Yi Jiang, JinTao Qian, Kai Li, Xuewen Zhu

**Affiliations:** ^1^ Department of Thoracic and Cardiovascular Surgery, Affiliated Drum Tower Hospital of Nanjing University Medical School, Institute of Cardiothoracic Vascular Disease Nanjing University Nanjing China; ^2^ Department of Anesthesiology, Nanjing Drum Tower Hospital The Affiliated Hospital of Nanjing University Medical School Nanjing China

**Keywords:** antioxidants, machine learning interpretation, NHANES, oxidative balance score, peripheral artery disease

## Abstract

Oxidative stress plays a central role in the development of peripheral artery disease (PAD), yet composite indices quantifying its impact remain underutilized. The oxidative balance score (OBS), integrating dietary and lifestyle exposures, may offer a comprehensive approach to vascular risk stratification. We analyzed data from 7249 U.S. adults in NHANES 1999–2004 to examine the association between OBS and PAD, defined by an ankle‐brachial index ≤ 0.9. Multivariable logistic regression and restricted cubic spline models were used to assess both linear and nonlinear associations. Twelve machine learning models were constructed to predict PAD status; the top‐performing model (GLMNet) was interpreted using SHapley Additive exPlanations (SHAP) to identify key predictors. Higher OBS levels were significantly associated with reduced PAD prevalence (fully adjusted OR per unit increase: 0.963; 95% CI: 0.944–0.983; *p* < 0.001), with a U‐shaped dose–response curve. The inverse association remained consistent across most subgroups. In sensitivity analyses focusing on diabetic individuals, the total OBS was not significantly associated with PAD, but the lifestyle component of OBS remained protective. SHAP analysis identified total folate intake, physical activity, and serum cotinine as the most influential predictors, while classic antioxidants such as vitamin C and vitamin E had limited predictive value. Integrating conventional regression and interpretable machine learning, this study demonstrates a nonlinear, inverse association between oxidative balance and PAD. OBS may serve as a practical composite biomarker for vascular risk profiling, with particular relevance for lifestyle‐based interventions in high‐risk populations.

## Graphical abstract

Higher oxidative balance score (OBS) is associated with lower PAD prevalence. SHAP analysis highlights folate, activity, and cotinine as top OBS predictors.

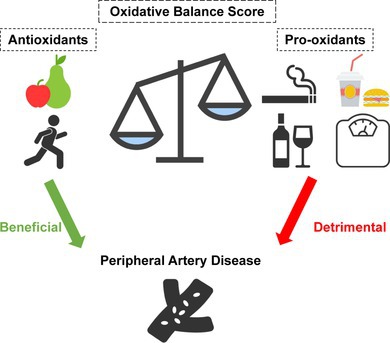

## 1 Introduction

Peripheral artery disease (PAD) is a chronic atherosclerotic condition characterized by the narrowing or occlusion of lower limb arteries, which affects over 200 million individuals globally and constitutes a significant public health concern due to its strong association with disability, cardiovascular events, and mortality (Aday and Matsushita 2021). While conventional risk factors—including hypertension, diabetes, smoking, and hyperlipidemia—are well‐established, growing evidence indicates that oxidative stress plays a fundamental role in PAD pathogenesis (GBD 2019 Peripheral Artery Disease Collaborators 2023; You et al. 2023). Oxidative stress, defined as an imbalance between reactive oxygen species (ROS) production and the endogenous antioxidant defense system, is widely considered a central driver of atherosclerosis, endothelial dysfunction, and the initiation of inflammatory cascades, all of which play a pivotal role in PAD pathology (Steven et al. 2017). The oxidative‐antioxidative balance, shaped by dietary habits, behavioral patterns, and metabolic conditions, is believed to be a key pathway in vascular aging, intimal thickening, and arterial sclerosis progression (Gabriele and Pucci 2017; Niemann et al. 2017). As a result, quantifying individual oxidative stress exposure has become an innovative but methodologically challenging focus in cardiovascular epidemiology.

To comprehensively assess the relationship between oxidative load and disease risk, the Oxidative Balance Score (OBS) was developed as an integrative index that reflects overall pro‐oxidant and antioxidant exposure derived from dietary habits, lifestyle behaviors, and nutritional biomarkers (Lei et al. 2023; Zhang et al. 2022). The OBS combines a range of antioxidant elements (e.g., vitamins C and E, carotenoids, polyphenols, minerals) and pro‐oxidant contributors (e.g., fat intake, smoking, and body mass index) into a unified scoring system, providing a comprehensive and temporally integrated measure of oxidative stress exposure. Although the OBS has demonstrated predictive validity in several chronic diseases such as metabolic syndrome (Wang and Shi 2025), diabetes (Kwon et al. 2023), and chronic kidney disease (Son et al. 2023), its application in PAD research remains limited, especially concerning the systematic evaluation of individual component contributions.

Concurrently, the growing dimensionality and complexity of biomedical data has rendered traditional regression models increasingly inadequate for capturing multifactorial interactions. Machine learning techniques have emerged as powerful tools in biomedical modeling by enabling nonlinear feature analysis and the identification of higher‐order interactions (Cao and Hu 2024). Interpretability frameworks such as SHapley Additive exPlanations (SHAP) enhance model transparency by allowing for quantitative attribution of individual predictor effects within multivariate models (Ponce‐Bobadilla et al. 2024). Integrating these approaches into OBS analysis enables identification of the most clinically informative components for PAD risk prediction within the oxidative stress framework as well as baseline clinical information.

This study aims to systematically investigate the association between OBS and PAD using nationally representative data from the U.S. National Health and Nutrition Examination Survey (NHANES), and to further quantify the relative contributions of individual OBS components to PAD risk via multiple machine learning models and SHAP analysis. Ultimately, the study seeks to address the current evidence gap in oxidative stress‐based vascular risk prediction and to inform future nutritional strategies for PAD prevention.

## 2 Materials and Methods

### 2.1 Data Source and Study Population

This study drew upon data from three consecutive NHANES cycles (1999–2004), which employed a stratified, multistage probability sampling design to produce a nationally representative U.S. population sample. The survey provides extensive and standardized data on sociodemographic characteristics, dietary intake, physical examinations, laboratory biomarkers, and self‐reported health conditions, making it a widely utilized resource in population‐based research on chronic diseases. The analytical sample was constructed by initially including all participants (*n* = 31,126), followed by sequential exclusion of individuals missing Ankle‐Brachial Index (ABI) data (*n* = 23,555), pregnant individuals (*n* = 7), and participants lacking data required for calculating OBS (*n* = 315). Ultimately, 7249 participants met the inclusion criteria and were retained for the final analysis. All participants provided written informed consent. The NHANES study protocol was approved by the National Center for Health Statistics (NCHS) Research Ethics Review Board, and no additional ethical approval was required for this secondary analysis.

### 2.2 Outcome Definition

The primary outcome of this study was PAD, defined using ABI (Aboyans et al. 2012). ABI is calculated as the ratio of systolic blood pressure at the ankle to that at the brachial artery, reflecting peripheral vascular resistance and perfusion. Participants rested in a supine position for several minutes before trained technicians measured systolic blood pressure at both posterior tibial arteries and the brachial artery using a standard cuff. ABI was determined as the ratio of the lower ankle systolic pressure to the higher brachial pressure. PAD was defined as an ABI ≤ 0.9, following established clinical guidelines.

### 2.3 Definition and Calculation of OBS

The OBS provides a comprehensive measure of an individual's cumulative exposure to pro‐oxidant and antioxidant factors derived from diet and lifestyle. In accordance with established literature, the OBS was constructed from 20 components, which were categorized into dietary and lifestyle domains (Lei et al. 2023; Zhang et al. 2022). The dietary domain encompassed 16 nutritional components: dietary fiber, carotenoids, riboflavin (vitamin B2), niacin, vitamin B6, vitamin B12, total folate, vitamins C and E, calcium, magnesium, zinc, selenium, copper, iron, and total fat intake. The lifestyle domain consisted of four components: body mass index (BMI), alcohol consumption, serum cotinine (as a biomarker for tobacco exposure), and total physical activity, which was quantified by metabolic equivalents (METs).

Each antioxidant‐related component was assigned a score based on tertile distributions, with values of 2, 1, and 0 corresponding to high, medium, and low levels of intake, respectively. Pro‐oxidant components were reverse‐coded using the same criteria. The total OBS was calculated by summing the individual component scores, where higher values indicate greater antioxidant predominance. Dietary intake data were obtained through 24‐h recall interviews conducted by trained personnel. For the 2003–2004 cycle, nutrient intake was estimated as the average of two recall days.

### 2.4 Covariate Definition and Measurement

To minimize confounding bias, a comprehensive set of covariates encompassing demographic characteristics, lifestyle behaviors, and clinical conditions was adjusted for in the analysis. Demographic variables included sex, age, race/ethnicity, marital status, educational attainment, and poverty income ratio (PIR). Body composition was assessed using BMI and categorized as normal (< 25 kg/m^2^) or overweight/obese (≥ 25 kg/m^2^). Relevant clinical comorbidities were also incorporated. Hypertension was defined as a mean systolic blood pressure ≥ 140 mmHg, a mean diastolic pressure ≥ 90 mmHg, a self‐reported diagnosis, or the use of antihypertensive medication. Diabetes was defined as a self‐reported physician diagnosis, HbA1c > 6.5%, fasting plasma glucose ≥ 7.0 mmol/L, random glucose ≥ 11.1 mmol/L, 2‐h oral glucose tolerance test (OGTT) glucose ≥ 11.1 mmol/L, or current use of glucose‐lowering medication (American Diabetes Association Professional Practice Committee 2024). Hyperlipidemia was defined as triglycerides ≥ 150 mg/dL, total cholesterol ≥ 200 mg/dL, LDL‐C ≥ 130 mg/dL, HDL‐C ≤ 40 mg/dL for men or ≤ 50 mg/dL for women, or use of lipid‐lowering therapy (National Cholesterol Education Program (NCEP) Expert Panel on Detection, Evaluation, and Treatment of High Blood Cholesterol in Adults (Adult Treatment Panel III) 2002). Atherosclerotic cardiovascular disease (ASCVD) was defined as a self‐reported history of coronary heart disease, angina, myocardial infarction, or stroke. Chronic kidney disease (CKD) was defined as an estimated glomerular filtration rate (eGFR) < 59 mL/min/1.73 m^2^ or a urinary albumin‐to‐creatinine ratio (ACR) > 30 mg/g (Rovin et al. 2021).

### 2.5 Feature Engineering and Machine Learning Modeling

The initial modeling process incorporated 37 candidate variables. To improve model stability and generalizability, variables exhibiting near‐zero variance—defined as those with a unique value frequency below 5%—were excluded. Next, Pearson correlation coefficients were computed; from each pair with *r* > 0.8, one variable was removed to reduce multicollinearity (Qi et al. 2025). Remaining variables were standardized using min–max normalization to ensure consistent scaling and prevent dominance by high‐magnitude features. Ultimately, 32 variables—including demographic characteristics, laboratory markers, and individual OBS components—were retained as model input features.

Twelve commonly used supervised machine learning algorithms were implemented using the R‐based mlr3 framework. These included GLMNet, Random Forest, Support Vector Machine (SVM), XGBoost, LightGBM, CatBoost, Naïve Bayes, k‐Nearest Neighbors (KNN), GBM, and GLMBoost. All models were trained with default hyperparameters and evaluated via five‐fold cross‐validation for PAD case classification. Performance metrics included the area under the receiver operating characteristic curve (AUC), accuracy, recall, precision, and F1 score.

### 2.6 Model Interpretability and SHAP Analysis

To enhance clinical interpretability, SHAP analysis was applied to quantify the marginal contribution of each predictor to PAD risk. SHAP, grounded in cooperative game theory, assigns consistent and equitable contribution values to each feature and is widely regarded as one of the most interpretable techniques for explaining black‐box models (Lundberg and Lee 2017; Nordin et al. 2023). The KernelSHAP algorithm was employed to interpret the GLMNet model. A background dataset of 200 randomly sampled training observations was used to estimate the effect of feature perturbations on prediction probabilities. The interpretive outputs comprised global feature importance rankings, SHAP beeswarm plots, and individual‐level waterfall plots, offering detailed visualization of the relationships between predictors and model‐predicted PAD risk.

### 2.7 Statistical Analysis

Analyses of NHANES data accounted for the complex survey design, incorporating sample weights, stratification, and clustering. Continuous variables were reported as means with standard deviations (SDs), and categorical variables as frequencies and proportions. Weighted logistic regression was employed to estimate odds ratios (ORs) and 95% confidence intervals (CIs) for PAD risk. A stepwise adjustment approach was applied across nested models. Model 1 included demographic covariates: race/ethnicity, age, sex, educational attainment, and marital status. Model 2 additionally adjusted for clinical comorbidities: hypertension, hyperlipidemia, diabetes, and ASCVD.

Restricted cubic splines (RCS) were applied to evaluate and visualize the dose–response relationship between OBS and PAD, with knots placed at the 10th, 50th, and 90th percentiles. Stratified and interaction analyses were conducted across key subgroups—including age, sex, BMI, diabetes, hypertension, dyslipidemia, and ASCVD—to explore potential effect modification.

To explore potential heterogeneity in the diabetic subgroup, OBS was partitioned into dietary and lifestyle domains, reflecting nutritional intake and behavioral factors, respectively. The association of each domain with PAD risk was evaluated using logistic regression and RCS analyses, with exposures modeled both continuously and by quartiles. Covariate adjustments were consistent with those used in the primary models.

All statistical analyses were performed using R software (version 4.4.3), incorporating the packages: survey, mlr3, mlr3benchmark, mlr3extralearner, kernelshap, and shapviz. A two‐sided *p*‐value < 0.05 was considered statistically significant.

## 3 Result

### 3.1 Study Population Characteristics

The NHANES cohort included 7249 participants, of whom 554 had PAD and 6695 were without PAD (Table 1). The mean age of the total sample was 56.28 ± 0.24 years; 51.93% were female, and 78.08% identified as White. Compared with individuals without PAD, those with PAD were generally older, more likely to be female, and had higher systolic but lower diastolic blood pressure. They also had lower levels of educational attainment and a higher prevalence of former smoking. Additionally, the burden of chronic diseases—including hypertension, diabetes, hyperlipidemia, ASCVD, and CKD—was substantially greater among participants with PAD.

**TABLE 1:** Baseline characteristics of NHANES participants.

	Total (*n* = 7249)	Without PAD (*n* = 6695)	With PAD (*n* = 554)	*p*
Age, years, mean (SD)	56.28 ± 0.24	55.66 ± 0.22	67.59 ± 0.68	< 0.0001
Sex, *n* (%)				0.07
Female	3577 (51.93)	3298 (51.65)	279 (57.09)	
Male	3672 (48.07)	3397 (48.35)	275 (42.91)	
Ethnicity, *n* (%)				< 0.0001
White	3959 (78.08)	3646 (78.04)	313 (78.84)	
Black	1282 (8.95)	1154 (8.68)	128 (13.99)	
Mexican	1520 (4.50)	1428 (4.55)	92 (3.56)	
Other	488 (8.47)	467 (8.73)	21 (3.61)	
Smoke status, *n* (%)				< 0.0001
Married or living with a partner	4624 (68.02)	4333 (71.31)	291 (58.83)	
Not married nor living with a partner	2375 (28.24)	2131 (28.69)	244 (41.17)	
Education, *n* (%)				< 0.0001
Below high school	2420 (19.71)	2186 (19.10)	234 (31.41)	
High school and above	4815 (80.17)	4497 (80.90)	318 (68.59)	
Smoke, *n* (%)				< 0.0001
Former	2494 (33.34)	2254 (32.89)	240 (42.09)	
Never	3366 (46.34)	3179 (47.13)	187 (32.57)	
Now	1379 (20.24)	1253 (19.98)	126 (25.34)	
Alcohol user, *n* (%)				< 0.0001
Never	1045 (12.28)	950 (12.23)	95 (16.91)	
Former	1790 (21.47)	1602 (21.22)	188 (32.44)	
Heavy	930 (13.08)	885 (13.51)	45 (9.10)	
Mild	2509 (37.85)	2340 (38.72)	169 (32.82)	
Moderate	848 (13.82)	805 (14.32)	43 (8.73)	
BMI, kg/m^2^, mean (SD)	28.34 ± 0.12	28.33 ± 0.13	28.59 ± 0.34	0.46
HbA1c, %, mean (SD)	5.62 ± 0.02	5.60 ± 0.02	5.93 ± 0.06	< 0.0001
SBP, mmHg, mean (SD)	127.94 ± 0.40	127.31 ± 0.41	139.59 ± 1.37	< 0.0001
DBP, mmHg, mean (SD)	73.79 ± 0.24	74.10 ± 0.25	68.10 ± 0.75	< 0.0001
TG, mmol/L, mean (SD)	1.84 ± 0.05	1.83 ± 0.05	1.95 ± 0.10	0.3
HDL, mmol/L, mean (SD)	1.37 ± 0.01	1.38 ± 0.01	1.34 ± 0.03	0.21
LDL, mmol/L, mean (SD)	3.26 ± 0.03	3.27 ± 0.03	3.10 ± 0.07	0.04
OBS, score, mean (SD)	18.95 ± 0.21	19.08 ± 0.22	16.55 ± 0.39	< 0.0001
Hypertension, *n* (%)	3958 (47.47)	3528 (45.99)	430 (74.98)	< 0.0001
DM, *n* (%)	1284 (13.01)	1122 (12.32)	162 (25.85)	< 0.0001
Hyperlipidemia, *n* (%)	5669 (78.17)	5217 (77.85)	452 (84.14)	0.002
ASCVD, *n* (%)	1051 (11.80)	876 (10.69)	175 (32.12)	< 0.0001
CKD, *n* (%)	1600 (16.28)	1341 (15.37)	259 (44.92)	< 0.0001

### 3.2 Association Between OBS and PAD

A significant inverse relationship was observed between OBS and the risk of PAD. When included as a continuous variable in logistic regression analysis, each one‐unit increase in OBS was associated with a significantly lower risk of PAD. In the crude (unadjusted) model, this inverse association was statistically significant (OR = 0.949; 95% CI: 0.933–0.966; *p* < 0.0001). After adjusting for demographic covariates—race/ethnicity, age, sex, educational attainment, and marital status (Model 1)—the association remained robust (OR = 0.958; 95% CI: 0.941–0.975; *p* < 0.0001). Further adjustment for clinical comorbidities (Model 2) yielded consistent results (OR = 0.963; 95% CI: 0.944–0.983; *p* < 0.001), confirming the stability of the association.

Categorization of OBS into quartiles revealed a stepwise decrease in PAD risk across higher quartiles (Table 2). Compared to participants in the lowest quartile (Q1), those in higher quartiles demonstrated progressively reduced odds of PAD. In the unadjusted model, the odds ratios for Q2, Q3, and Q4 were 0.524 (95% CI: 0.388–0.707; *p* < 0.0001), 0.486 (95% CI: 0.367–0.642; *p* < 0.0001), and 0.396 (95% CI: 0.266–0.590; *p* < 0.0001), respectively, indicating a significant linear trend (*p* for trend < 0.0001). This inverse gradient persisted after demographic adjustment (Model 1), with the highest quartile (Q4) associated with a 52.1% reduced risk compared to Q1 (OR = 0.479; 95% CI: 0.328–0.699; *p* < 0.001; *p* for trend < 0.001). Although the association was modestly attenuated in the fully adjusted model (Model 2), it remained statistically significant (Q4 vs. Q1: OR = 0.531; 95% CI: 0.360–0.782; *p* = 0.002; *p* for trend = 0.001). Overall, these findings demonstrate a robust inverse association between OBS and PAD risk, regardless of whether OBS is analyzed as a continuous or categorical variable.

**TABLE 2:** Association between OBS and PAD.

	Crude model		Model 1		Model 2	
	HR (95% CI)	*p*	HR (95% CI)	*p*	HR (95% CI)	*p*
Continuous	0.949 (0.933, 0.966)	< 0.0001	0.958 (0.941, 0.975)	< 0.0001	0.963 (0.944, 0.983)	< 0.001
Q1	ref		ref		ref	
Q2	0.524 (0.388, 0.707)	< 0.0001	0.523 (0.376, 0.727)	< 0.001	0.503 (0.354, 0.713)	< 0.001
Q3	0.486 (0.367, 0.642)	< 0.0001	0.554 (0.402, 0.765)	< 0.001	0.537 (0.384, 0.753)	< 0.001
Q4	0.396 (0.266, 0.590)	< 0.0001	0.479 (0.328, 0.699)	< 0.001	0.531 (0.360, 0.782)	0.002
*p* for trend		< 0.0001		< 0.001		0.001

*Note:* Crude model: unadjusted for none. Model 1 adjusted for: ethnicity, age, sex, education, marital status. Model 2 adjusted for: ethnicity, age, sex, education, marital status, hypertension, hyperlipidemia, atherosclerotic cardiovascular disease, diabetes, chronic kidney disease.

### 3.3 Nonlinear Dose–Response Relationship Between OBS and PAD

**FIGURE 1:**
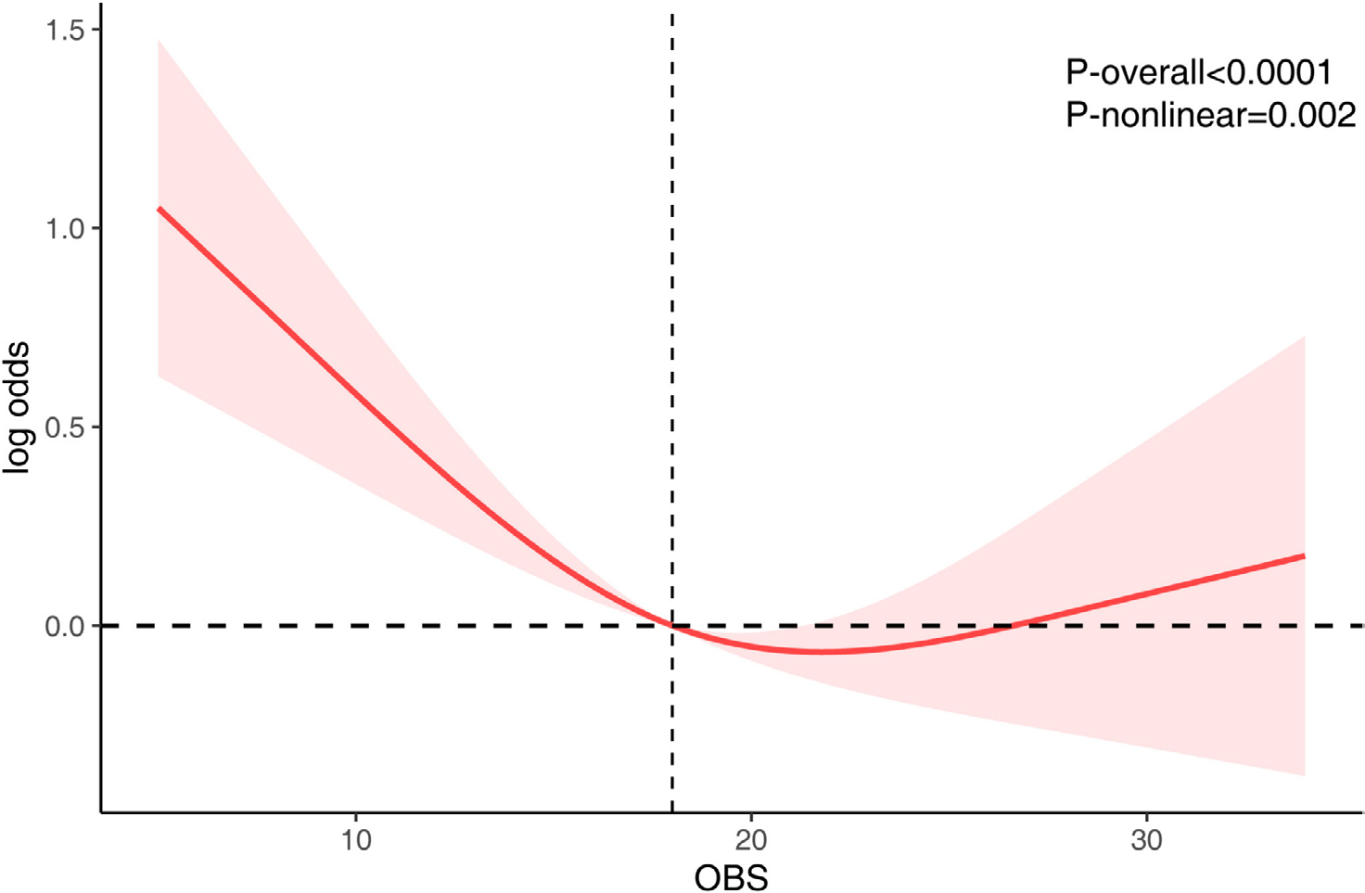
The RCS analysis between OBS and PAD. The model was adjusted for ethnicity, age, sex, education, marital status, hypertension, hyperlipidemia, atherosclerotic cardiovascular disease, diabetes, chronic kidney disease.

Figure 1 presents the nonlinear dose–response association between the Oxidative Balance Score (OBS) and PAD risk, evaluated using an RCS model embedded within a weighted logistic regression framework. The analysis demonstrated a statistically significant overall and nonlinear association (*p*‐overall < 0.0001; *p*‐nonlinear = 0.002). As OBS increased, the log‐odds of PAD decreased steadily, reaching the lowest point of risk at an OBS value of approximately 21.76. This inflection suggests that the protective effect of oxidative balance is most evident up to this threshold. Beyond this point, the PAD risk curve showed a slight upward trend; although the overall risk remained relatively low, forming a U‐shaped trajectory.

### 3.4 Subgroup Analyses

**FIGURE 2:**
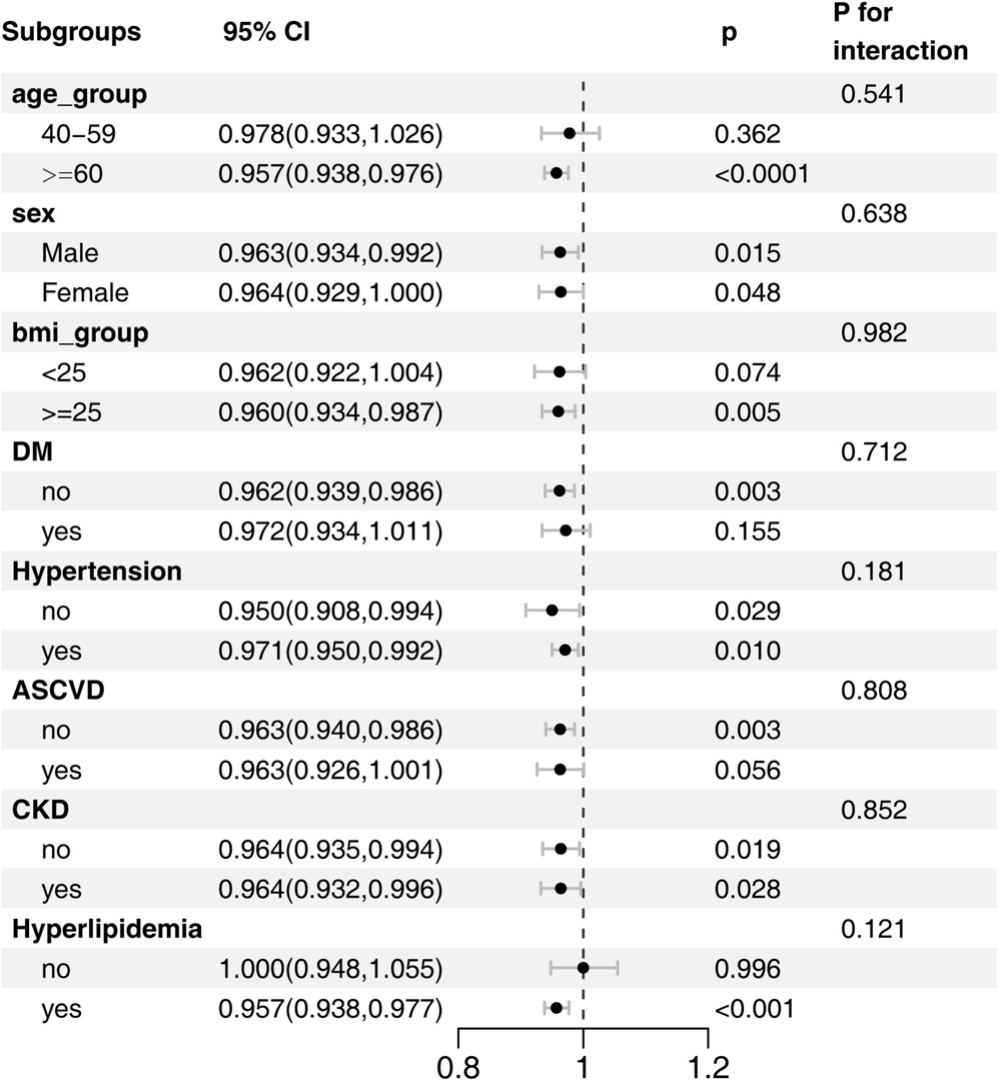
Subgroup analysis of the association between OBS and PAD. The model was adjusted for ethnicity, age, sex, education, marital status, hypertension, hyperlipidemia, atherosclerotic cardiovascular disease, diabetes, and chronic kidney disease.

To further evaluate the robustness and potential heterogeneity of the association between OBS and PAD, stratified analyses were conducted across subgroups defined by age, sex, BMI, diabetes, hypertension, ASCVD, CKD, and hyperlipidemia status (Figure 2). Across all stratified subgroups, the inverse association between OBS and PAD was generally consistent in direction and magnitude. Although no statistically significant interaction effects were observed (all *p* for interaction > 0.05), the pattern of similar effect estimates across subgroups suggests that the association may be relatively stable across diverse population strata.

For instance, significant inverse associations were found in both men (OR = 0.963; 95% CI: 0.934–0.992; *p* = 0.015) and women (OR = 0.964; 95% CI: 0.929–1.000; *p* = 0.048). Among participants with BMI ≥ 25 kg/m^2^, the association remained robust (OR = 0.960; 95% CI: 0.934–0.987; *p* = 0.005), while it approached statistical significance in those with BMI < 25 kg/m^2^ (*p* = 0.074). In non‐diabetic individuals, the inverse association was maintained (OR = 0.962; 95% CI: 0.939–0.986; *p* = 0.003); whereas no significant association was observed among those with diabetes (*p* = 0.155). Notably, the inverse association was particularly pronounced in participants with dyslipidemia (OR = 0.957; 95% CI: 0.938–0.977; *p* < 0.001); whereas no association was observed in those without hyperlipidemia (*p* = 0.996). However, the interaction between dyslipidemia status and OBS was not statistically significant (*p* for interaction = 0.121), suggesting that while the magnitude of the association varied, formal evidence of effect modification was lacking. These findings imply that the protective effect of oxidative balance may be more evident in metabolically vulnerable populations.

### 3.5 Sensitivity Analysis: OBS Dietary and Lifestyle Components in Diabetic Subgroup

To evaluate the separate contributions of oxidative balance domains among diabetic individuals, we partitioned OBS into dietary and lifestyle components and assessed their associations with PAD risk. In this subgroup, the total OBS was inversely associated with PAD in the crude model (OR, 0.960; 95% CI, 0.931–0.991), this relationship was attenuated and lost statistical significance in fully adjusted models (Model 2; OR, 0.973; 95% CI, 0.935–1.013; *p* = 0.180) (Table S1). A restricted cubic spline model indicated a significant nonlinear (U‐shaped) dose–response pattern (*p*‐overall = 0.0047; *p*‐nonlinearity = 0.005) (Figure S1).

In domain‐specific models, the lifestyle OBS was significantly associated with reduced PAD risk in fully adjusted models (OR, 0.797; 95% CI, 0.638–0.994); whereas, the dietary OBS showed no significant association (OR, 0.981; 95% CI, 0.942–1.022) (Table S2). Quartile‐based analyses further supported this pattern: participants in the highest quartile of the lifestyle OBS had a 79.1% lower odds of PAD compared to those in the lowest quartile (OR for Q4 vs. Q1: 0.209; 95% CI: 0.065–0.668; *p* for trend = 0.006) (Table S2). This association followed a predominantly linear pattern (*p*‐nonlinearity = 0.450) (Figure S2). In contrast, the dietary OBS showed a nonlinear (U‐shaped) association with PAD (*p*‐overall = 0.004; *p*‐nonlinearity = 0.002), and effect sizes were attenuated in multivariable models (Table S2, Figure S3). These findings suggest that lifestyle‐related oxidative exposures may play a more prominent role in PAD risk modulation among diabetic individuals than dietary factors alone.

### 3.6 Comparative Performance of Machine Learning Models

Table 3 summarizes the predictive performance of 12 supervised machine learning algorithms in classifying PAD cases within the NHANES cohort. All models were trained using five‐fold cross‐validation and evaluated across multiple performance metrics, including area under the receiver operating characteristic curve (AUC), accuracy, recall, precision, and F1 score.

**TABLE 3:** Performance comparison of machine learning models in predicting PAD.

Learner	AUC	ACC	Recall	Precision	F1 score
CatBoost	0.757	0.937	1.000	0.937	0.967
Support vector machine	0.643	0.936	1.000	0.936	0.967
Random forest for survival, regression and classification	0.748	0.936	1.000	0.936	0.967
GLMNet	0.768	0.936	1.000	0.936	0.967
Ranger	0.747	0.936	1.000	0.936	0.967
XGBoost	0.746	0.934	0.994	0.940	0.966
GBM	0.767	0.937	0.994	0.942	0.967
GLMBoost	0.757	0.936	1.000	0.936	0.967
LightGBM	0.761	0.936	0.998	0.938	0.967
Naïve Bayes	0.717	0.847	0.878	0.955	0.914
Random Forest	0.733	0.936	1.000	0.936	0.967
k‐Nearest Neighbors	0.599	0.931	0.993	0.937	0.964

**FIGURE 3:**
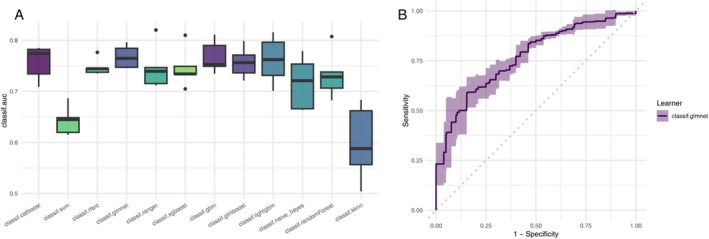
Comparative performance of machine learning models for PAD classification. (A) Boxplot of area under the ROC curve (AUC) across 12 supervised machine learning models using five‐fold cross‐validation. (B) Receiver operating characteristic (ROC) curve with 95% confidence interval for the best‐performing model (GLMNet).

Among all models, GLMNet—a generalized linear model with Lasso regularization—achieved the highest discriminative performance, with an AUC of 0.768, an accuracy of 93.6%, a perfect recall of 100%, and an F1 score of 0.967 (Figure 3). GBM demonstrated comparable performance, yielding the same AUC (0.767), a slightly higher accuracy (93.7%), a recall of 99.4%, a precision of 94.2%, and an F1 score of 0.967.

Other ensemble learning algorithms—including CatBoost, LightGBM, and Random Forest—also performed strongly, with AUCs exceeding 0.74 and recall rates close to or equal to 100%, indicating high sensitivity in detecting PAD cases. In contrast, KNN and SVM showed relatively weaker performance, with AUCs of 0.599 and 0.643, respectively. While recall remained high, their overall discriminative ability was inferior to that of the ensemble‐based models. Naïve Bayes achieved a high precision of 95.5%, yet it exhibited lower AUC and accuracy compared to the top‐performing models, suggesting limited generalizability and stability under the current modeling framework.

### 3.7 SHAP‐Based Feature Contribution and Importance Analysis

To evaluate the relative importance and directional impact of individual predictors in classifying PAD, we applied SHAP analysis based on the GLMNet model. The results indicated that age was the most influential variable (mean SHAP value = 0.028), followed by DBP, PIR, and total folate intake (mean SHAP = 0.0067). These four features comprised the top contributors in the model. Notably, total folate—an antioxidant‐related dietary component within the OBS—ranked fourth overall, underscoring its potential independent role in predicting PAD risk.

**FIGURE 4:**
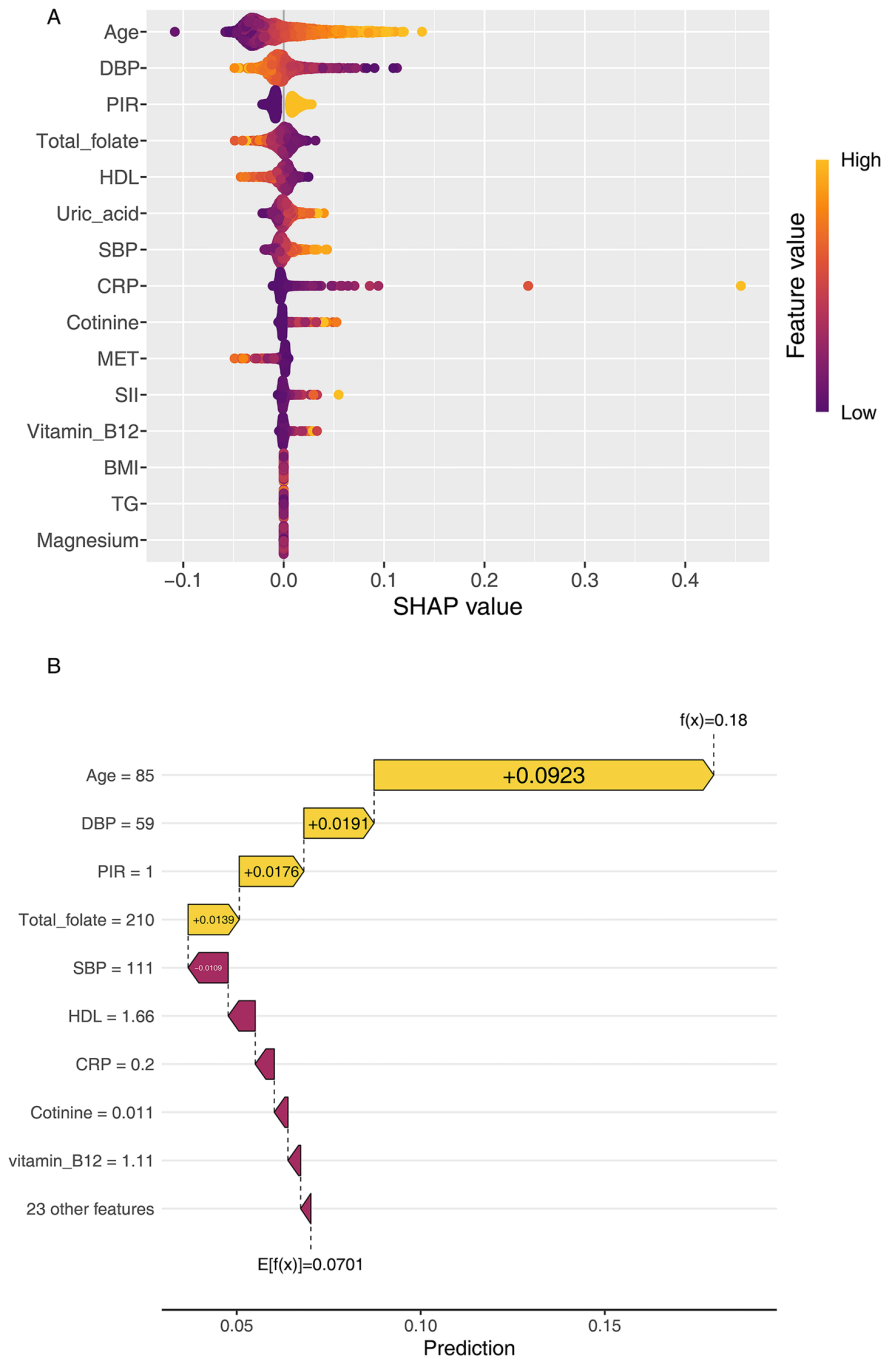
SHAP interpretation of the GLMNet model for PAD classification. (A) SHAP beeswarm plot showing the global importance and effects of key features. (B) SHAP waterfall plot illustrating how each feature contributed to an individual's predicted PAD risk.

The SHAP beeswarm plot (Figure 4A) revealed a clear inverse relationship between higher folate intake and PAD risk, supporting its potential protective effect on peripheral vascular health. Other OBS‐related predictors, such as serum cotinine (a biomarker of tobacco exposure) and total physical activity (measured in METs), also showed notable contributions to the model, highlighting the relevance of lifestyle‐related variables in modulating oxidative balance. In contrast, several dietary antioxidants included in the OBS—such as dietary fiber, vitamin C, vitamin E, and selenium—exhibited low SHAP values and were ranked lower in overall feature importance. These findings suggest that the influence of these specific nutrients on PAD risk prediction is limited in this population.

In addition, Figure 4B presents a SHAP waterfall plot for an individual prediction instance, illustrating how each feature incrementally contributes to the model's output probability for PAD. Age, DBP, PIR, and total folate intake collectively shifted the baseline prediction (expected value: 0.070) upward to a final predicted risk of 0.18. Features such as SBP, HDL, and CRP exerted smaller downward effects, attenuating the overall prediction.

## 4 Discussion

Leveraging data from the nationally representative NHANES cohort, this study systematically investigated the association between OBS and PAD, combining multivariable statistical models with interpretable machine learning approaches to evaluate the predictive utility of OBS. A significant inverse association was observed: individuals with higher OBS levels—indicating greater antioxidant exposure—had a lower likelihood of developing PAD. Restricted cubic spline modeling revealed a nonlinear, U‐shaped dose–response relationship, with the lowest estimated PAD risk occurring at moderate to moderately high OBS levels. Subgroup analyses confirmed the consistency of this association across most demographic and clinical strata. Further insights were obtained through GLMNet modeling and SHAP analysis, which identified total folate intake, physical activity, and serum cotinine (a biomarker of tobacco exposure) as the most influential contributors to PAD prediction. These findings support the potential use of OBS as an integrative biomarker for cardiovascular risk stratification and provide empirical evidence for addressing oxidative stress in PAD prevention strategies.

Oxidative stress has long been implicated as a central mechanism in atherosclerosis and PAD pathogenesis (Canugovi et al. 2019; Zhou et al. 2012). It contributes to vascular injury by promoting LDL oxidation (Surendran et al. 2019), impairing endothelial function (An et al. 2023), and activating inflammatory pathways (An et al. 2023), thereby accelerating arterial narrowing and intimal hyperplasia. While antioxidant supplementation—such as with vitamins C and E or carotenoids—has shown some vascular benefits in interventional studies (Li et al. 2021; Woolf et al. 2023), findings from randomized controlled trials remain inconsistent (Keramat et al. 2022). The lack of clear efficacy from single‐nutrient interventions may reflect the multifactorial nature of redox imbalance and its modulation by lifestyle factors (Bevan and White Solaru 2020; Bonaca et al. 2021). Behaviors such as smoking and alcohol consumption can exacerbate oxidative burden and diminish the potential benefits of isolated antioxidants (Matyas et al. 2016; Rezk‐Hanna et al. 2022). These findings suggest that focusing solely on individual antioxidant nutrients may be insufficient for addressing the complex oxidative mechanisms involved in PAD. A more effective approach may lie in evaluating and targeting the overall oxidative–antioxidative balance, as captured by composite indices such as the OBS.

Notably, our spline analysis indicated a U‐shaped association between OBS and PAD risk, with the lowest risk observed at an OBS around 21.76. This suggests that while moderate to moderately high antioxidant exposure is protective, excessive antioxidant intake may yield diminishing returns or even potential harm. This pattern aligns with prior findings in nutritional epidemiology (Anselmo and Driscoll 2021; Bjelakovic et al. 2014), where supra‐physiological levels of certain antioxidants, such as β‐carotene (Terao 2023) or folate (Fardous and Heydari 2023), have shown paradoxical effects, particularly among smokers (EFSA Panel on Nutrition, Novel Foods and Food Allergens (NDA), et al. 2024) or patients with preexisting disease (Moustakli et al. 2024). Potential explanations for this include pro‐oxidant effects at high concentrations, interference with endogenous antioxidant systems, or nutrient–nutrient interactions that offset benefits (Terao 2023). Thus, our findings highlight the need for a balanced approach to oxidative stress modulation, avoiding both deficiency and excess.

Importantly, the inverse association between OBS and PAD was attenuated and no longer significant among individuals with diabetes mellitus. This may reflect the elevated and chronic oxidative burden characteristic of diabetic pathophysiology (Lira‐Meriguete et al. 2024), which can overwhelm the protective effects of dietary and behavioral antioxidants. Diabetes entails persistent hyperglycemia, increased formation of advanced glycation end‐products (Khalid et al. 2022), and systemic inflammation (Weinberg Sibony et al. 2024)—all of which amplify oxidative stress and contribute to vascular dysfunction. Furthermore, nutrient absorption and bioavailability of key antioxidants such as B‐vitamins and carotenoids (Desmarchelier and Borel 2017; Lee et al. 2022; Uebanso et al. 2020) may be compromised in diabetic states, thereby limiting their effectiveness. Notably, the lifestyle component of OBS—encompassing physical activity, smoking, alcohol intake, and BMI—remained significantly associated with reduced PAD risk in this subgroup. These findings underscore the need for lifestyle‐based oxidative stress reduction strategies tailored to metabolically vulnerable populations such as individuals with diabetes (Wronka et al. 2022).

OBS captures both dietary and lifestyle contributors to oxidative–antioxidative status, incorporating antioxidants (e.g., carotenoids, vitamins C and E, selenium) alongside pro‐oxidative behaviors (e.g., high BMI, smoking, low physical activity). This integrative framework provides a more comprehensive reflection of long‐term oxidative exposure than analyses focusing on single nutrients alone (Hernández‐Ruiz et al. 2019; Zhang et al. 2022). Previous studies have demonstrated that higher OBS levels are associated with reduced risks of type 2 diabetes (Kwon et al. 2023), chronic kidney disease (Son et al. 2023), cardiovascular events (Cheng et al. 2023), and all‐cause mortality (Talavera‐Rodriguez et al. 2023). However, there has been limited direct evidence examining the association between OBS and PAD. Our analysis is the first to demonstrate, using nationally representative data, that elevated OBS levels are robustly associated with a lower likelihood of PAD—even after adjustment for major demographic and clinical covariates. The identified nonlinear association further suggests that moderate enhancements in antioxidant exposure may confer maximal benefit, whereas extremely high levels may offer diminishing returns—though this potential threshold effect warrants additional investigation. Compared to conventional single‐nutrient assessments, the OBS provides a physiologically relevant, behaviorally informed index of redox status that aligns with real‐world dietary and lifestyle patterns. In this context, OBS may serve as a practical and informative tool for identifying individuals at elevated vascular risk and guiding personalized nutrition‐based interventions for PAD and related chronic vascular conditions.

Despite the overall predictive utility of OBS as a composite index (Lassale et al. 2016; Nerurkar et al. 2021), our findings suggest that its individual components contribute unequally to PAD risk prediction. By applying SHAP analysis within the GLMNet framework, we quantified the marginal effects of each variable and observed notable heterogeneity in feature importance. Among the top predictors, total folate intake ranked fourth overall and consistently showed a protective effect. As a critical cofactor in homocysteine metabolism, folate plays a well‐established role in vascular protection by mitigating endothelial injury and oxidative stress (Huang et al. 2024; Xu et al. 2022). Similarly, physical activity—measured in METs—was among the most influential predictors, likely due to its impact on mitochondrial redox homeostasis, free radical suppression, and vascular function enhancement (Monserrat‐Mesquida et al. 2022). Cotinine, a metabolite of nicotine exposure, demonstrated a positive association with PAD risk, reinforcing the oxidative vascular damage associated with tobacco use (Kunutsor et al. 2018). Notably, serum uric acid—although not included in the OBS—emerged as an independent risk factor in our model. This aligns with the compound's dual biological properties: while it may exhibit antioxidant behavior at low concentrations, elevated levels (hyperuricemia) are considered markers of pro‐oxidative and pro‐atherogenic states (Zhao et al. 2025).

In contrast, several classic dietary antioxidants within the OBS—such as vitamin C and vitamin E—were associated with low SHAP values, suggesting limited predictive contribution in this cohort and analytical context. These discrepancies may reflect narrow intake ranges or interindividual variability in bioavailability (Jensen et al. 2021; McNulty et al. 2019). Collectively, these findings indicate that future optimization of OBS weighting schemes should consider integrating objective biomarkers to more accurately capture antioxidant status.

This study has several strengths. First, it utilized data from NHANES, a nationally representative survey, enhancing the generalizability and public health relevance of the findings. Second, the relationship between OBS and PAD was thoroughly assessed using a combination of multivariable‐adjusted regression models, restricted cubic spline analyses, and stratified interaction assessments, ensuring the robustness of the observed associations. Third, a wide range of supervised machine learning algorithms were employed, and the application of SHAP provided an interpretable framework to evaluate individual variable contributions. This methodological approach not only improved model transparency but also enabled the identification of potentially modifiable targets.

Nevertheless, several limitations must be acknowledged. First, the cross‐sectional design of NHANES limits our ability to infer causality between OBS and PAD; longitudinal cohort studies are needed to establish temporal relationships. Second, the construction of OBS relied on 24‐h dietary recalls and self‐reported behavioral data, which may introduce recall bias and measurement error. Third, despite efforts to reduce multicollinearity during variable selection, residual intercorrelation among OBS components may still influence the stability and interpretation of feature contributions. Moreover, fat‐soluble antioxidants such as vitamins A, D, and E may have been underrepresented due to individual differences in absorption and metabolic utilization (Borel and Desmarchelier 2018). Finally, as the sample primarily consisted of middle‐aged and older adults in the United States, caution is warranted in generalizing the findings to other racial, ethnic, or dietary populations.

## 5 Conclusion

In this nationally representative study, a significant inverse association was observed between OBS and the risk of PAD. By integrating traditional epidemiologic methods with SHAP‐interpretable machine learning models, we demonstrated that higher OBS levels were linked to lower PAD prevalence, with a nonlinear, U‐shaped dose–response relationship. SHAP analysis identified total folate intake, physical activity, and nicotine exposure as the most influential predictors. These findings highlight the relevance of oxidative balance in PAD pathogenesis and suggest that OBS may serve as a practical composite marker for vascular risk assessment. Further longitudinal studies are needed to validate these results and explore the clinical application of OBS in cardiovascular prevention strategies.

## Author Contributions

**Zeyi Zhou:** conceptualization (lead), data curation (lead), formal analysis (equal), methodology (equal), software (equal), validation (equal), visualization (equal), writing – original draft (lead), writing – review and editing (equal). **Han Lee:** conceptualization (equal), data curation (supporting), methodology (supporting), software (supporting), visualization (supporting), writing – original draft (equal). **Yi Jiang:** data curation (supporting), methodology (supporting), software (supporting), writing – original draft (supporting). **JinTao Qian:** investigation (supporting), methodology (supporting), software (supporting), writing – original draft (supporting). **Kai Li:** formal analysis (equal), project administration (equal), writing – original draft (equal), writing – review and editing (lead). **Xuewen Zhu:** conceptualization (lead), methodology (equal), project administration (lead), visualization (equal), writing – original draft (equal), writing – review and editing (lead).

## Ethics Statement

The study protocol for NHANES underwent review and approval by the NCHS Research Ethics Review Committee; all participants provided written informed consent.

## Consent

The authors have nothing to report.

## Conflicts of Interest

The authors declare no conflicts of interest.

## Supporting information


**Data S1:** fsn370798‐sup‐0001‐Supinfo.pdf.

## Data Availability

The data derived from the National Health and Nutrition Examination Survey can be publicly accessed at https://wwwn.cdc.gov/nchs/nhanes/Default.aspx.

## References

[fsn370798-bib-0001] Aboyans, V. , M. H. Criqui , P. Abraham , et al. 2012. “Measurement and Interpretation of the Ankle‐Brachial Index: A Scientific Statement From the American Heart Association.” Circulation 126, no. 24: 2890–2909. 10.1161/CIR.0b013e318276fbcb. 23159553

[fsn370798-bib-0002] Aday, A. W. , and K. Matsushita . 2021. “Epidemiology of Peripheral Artery Disease and Polyvascular Disease.” Circulation Research 128, no. 12: 1818–1832. 10.1161/CIRCRESAHA.121.318535. 34110907 PMC8202714

[fsn370798-bib-0003] American Diabetes Association Professional Practice Committee . 2024. “2. Diagnosis and Classification of Diabetes: Standards of Care in Diabetes‐2024.” Diabetes Care 47, no. Suppl 1: S20–S42. 10.2337/dc24-S002. 38078589 PMC10725812

[fsn370798-bib-0004] An, Y. , B.‐T. Xu , S.‐R. Wan , et al. 2023. “The Role of Oxidative Stress in Diabetes Mellitus‐Induced Vascular Endothelial Dysfunction.” Cardiovascular Diabetology 22, no. 1: 237. 10.1186/s12933-023-01965-7. 37660030 PMC10475205

[fsn370798-bib-0005] Anselmo, F. , and M. S. Driscoll . 2021. “Deleterious Side Effects of Nutritional Supplements.” Clinics in Dermatology 39, no. 5: 745–756. 10.1016/j.clindermatol.2021.05.002. 34785002

[fsn370798-bib-0006] Bevan, G. H. , and K. T. White Solaru . 2020. “Evidence‐Based Medical Management of Peripheral Artery Disease.” Arteriosclerosis, Thrombosis, and Vascular Biology 40, no. 3: 541–553. 10.1161/ATVBAHA.119.312142. 31996023

[fsn370798-bib-0007] Bjelakovic, G. , D. Nikolova , and C. Gluud . 2014. “Antioxidant Supplements and Mortality.” Current Opinion in Clinical Nutrition and Metabolic Care 17, no. 1: 40–44. 10.1097/MCO.0000000000000009. 24241129

[fsn370798-bib-0008] Bonaca, M. P. , N. M. Hamburg , and M. A. Creager . 2021. “Contemporary Medical Management of Peripheral Artery Disease.” Circulation Research 128, no. 12: 1868–1884. 10.1161/CIRCRESAHA.121.318258. 34110910

[fsn370798-bib-0009] Borel, P. , and C. Desmarchelier . 2018. “Bioavailability of Fat‐Soluble Vitamins and Phytochemicals in Humans: Effects of Genetic Variation.” Annual Review of Nutrition 38: 69–96. 10.1146/annurev-nutr-082117-051628. 30130464

[fsn370798-bib-0010] Canugovi, C. , M. D. Stevenson , A. E. Vendrov , et al. 2019. “Increased Mitochondrial NADPH Oxidase 4 (NOX4) Expression in Aging Is a Causative Factor in Aortic Stiffening.” Redox Biology 26: 101288. 10.1016/j.redox.2019.101288. 31419754 PMC6831838

[fsn370798-bib-0011] Cao, S. , and Y. Hu . 2024. “Creating Machine Learning Models That Interpretably Link Systemic Inflammatory Index, Sex Steroid Hormones, and Dietary Antioxidants to Identify Gout Using the SHAP (SHapley Additive exPlanations) Method.” Frontiers in Immunology 15: 1367340. 10.3389/fimmu.2024.1367340. 38751428 PMC11094226

[fsn370798-bib-0012] Cheng, S. , Y. Han , L. Jiang , Z. Lan , H. Liao , and J. Guo . 2023. “Associations of Oxidative Balance Score and Visceral Adiposity Index With Risk of Ischaemic Heart Disease: A Cross‐Sectional Study of NHANES, 2005‐2018.” BMJ Open 13, no. 7: e072334. 10.1136/bmjopen-2023-072334. PMC10351262 37451720

[fsn370798-bib-0013] Desmarchelier, C. , and P. Borel . 2017. “Overview of Carotenoid Bioavailability Determinants: From Dietary Factors to Host Genetic Variations.” Trends in Food Science & Technology 69: 270–280. 10.1016/j.tifs.2017.03.002.

[fsn370798-bib-0014] EFSA Panel on Nutrition, Novel Foods and Food Allergens (NDA) , D. Turck , T. Bohn , et al. 2024. “Scientific Opinion on the Tolerable Upper Intake Level for Preformed Vitamin A and β‐Carotene.” EFSA Journal European Food Safety Authority 22, no. 6: e8814. 10.2903/j.efsa.2024.8814. 38846679 PMC11154838

[fsn370798-bib-0015] Fardous, A. M. , and A. R. Heydari . 2023. “Uncovering the Hidden Dangers and Molecular Mechanisms of Excess Folate: A Narrative Review.” Nutrients 15, no. 21: 4699. 10.3390/nu15214699. 37960352 PMC10648405

[fsn370798-bib-0016] Gabriele, M. , and L. Pucci . 2017. “Diet Bioactive Compounds: Implications for Oxidative Stress and Inflammation in the Vascular System.” Endocrine, Metabolic & Immune Disorders Drug Targets 17, no. 4: 264–275. 10.2174/1871530317666170921142055. 28933306

[fsn370798-bib-0017] GBD 2019 Peripheral Artery Disease Collaborators . 2023. “Global Burden of Peripheral Artery Disease and Its Risk Factors, 1990‐2019: A Systematic Analysis for the Global Burden of Disease Study 2019.” Lancet. Global Health 11, no. 10: e1553–e1565. 10.1016/S2214-109X(23)00355-8. 37734799 PMC10522777

[fsn370798-bib-0018] Hernández‐Ruiz, Á. , B. García‐Villanova , E. Guerra‐Hernández , P. Amiano , M. Ruiz‐Canela , and E. Molina‐Montes . 2019. “A Review of A Priori Defined Oxidative Balance Scores Relative to Their Components and Impact on Health Outcomes.” Nutrients 11, no. 4: 774. 10.3390/nu11040774. 30987200 PMC6520884

[fsn370798-bib-0019] Huang, X. , H. Bao , C. Ding , et al. 2024. “Optimal Folic Acid Dosage in Lowering Homocysteine: Precision Folic Acid Trial to Lower Homocysteine (PFAT‐Hcy).” European Journal of Nutrition 63, no. 5: 1513–1528. 10.1007/s00394-024-03344-8. 38478042 PMC11329420

[fsn370798-bib-0020] Jensen, M. B. , A. Daugintis , and J. Jakobsen . 2021. “Content and Bioaccessibility of Vitamin K (Phylloquinone and Menaquinones) in Cheese.” Foods (Basel, Switzerland) 10, no. 12: 2938. 10.3390/foods10122938. 34945489 PMC8700448

[fsn370798-bib-0021] Keramat, S. , H. Sharebiani , M. Patel , B. Fazeli , and A. Stanek . 2022. “The Potential Role of Antioxidants in the Treatment of Peripheral Arterial Disease: A Systematic Review.” Antioxidants 11, no. 11: 2126. 10.3390/antiox11112126. 36358498 PMC9686635

[fsn370798-bib-0022] Khalid, M. , G. Petroianu , and A. Adem . 2022. “Advanced Glycation End Products and Diabetes Mellitus: Mechanisms and Perspectives.” Biomolecules 12, no. 4: 542. 10.3390/biom12040542. 35454131 PMC9030615

[fsn370798-bib-0023] Kunutsor, S. K. , J. M. Spee , L. M. Kieneker , et al. 2018. “Self‐Reported Smoking, Urine Cotinine, and Risk of Cardiovascular Disease: Findings From the PREVEND (Prevention of Renal and Vascular End‐Stage Disease) Prospective Cohort Study.” Journal of the American Heart Association 7, no. 10: e008726. 10.1161/JAHA.118.008726. 29720504 PMC6015309

[fsn370798-bib-0024] Kwon, Y.‐J. , H.‐M. Park , and J.‐H. Lee . 2023. “Inverse Association Between Oxidative Balance Score and Incident Type 2 Diabetes Mellitus.” Nutrients 15, no. 11: 2497. 10.3390/nu15112497. 37299460 PMC10255164

[fsn370798-bib-0025] Lassale, C. , K. Castetbon , F. Laporte , et al. 2016. “Correlations Between Fruit, Vegetables, Fish, Vitamins, and Fatty Acids Estimated by Web‐Based Nonconsecutive Dietary Records and Respective Biomarkers of Nutritional Status.” Journal of the Academy of Nutrition and Dietetics 116, no. 3: 427–438.e5. 10.1016/j.jand.2015.09.017. 26522988

[fsn370798-bib-0026] Lee, S. Y. , D. Y. Lee , J. H. Kang , et al. 2022. “Effect of Age‐Related In Vitro Human Digestion With Gut Microbiota on Antioxidative Activity and Stability of Vitamins.” LWT 159: 113243. 10.1016/j.lwt.2022.113243.

[fsn370798-bib-0027] Lei, X. , Z. Xu , and W. Chen . 2023. “Association of Oxidative Balance Score With Sleep Quality: NHANES 2007‐2014.” Journal of Affective Disorders 339: 435–442. 10.1016/j.jad.2023.07.040. 37442450

[fsn370798-bib-0028] Li, W. , J. Yu , J. Zhao , et al. 2021. “Poria Cocos Polysaccharides Reduces High‐Fat Diet‐Induced Arteriosclerosis in ApoE−/− Mice by Inhibiting Inflammation.” Phytotherapy Research: PTR 35, no. 4: 2220–2229. 10.1002/ptr.6980. 33350533

[fsn370798-bib-0029] Lira‐Meriguete, A. M. , M. P. Santos , V. C. S. Viana , et al. 2024. “Can Pharmaceutical Care Decrease the Oxidative Stress in Type 2 Diabetes Mellitus?” Biomedicine & Pharmacotherapy = Biomedecine & Pharmacotherapie 171: 116178. 10.1016/j.biopha.2024.116178. 38266624

[fsn370798-bib-0030] Lundberg, S. , and S.‐I. Lee . 2017. “A Unified Approach to Interpreting Model Predictions (No. arXiv:1705.07874).” arXiv. 10.48550/arXiv.1705.07874.

[fsn370798-bib-0031] Matyas, C. , Z. V. Varga , P. Mukhopadhyay , et al. 2016. “Chronic Plus Binge Ethanol Feeding Induces Myocardial Oxidative Stress, Mitochondrial and Cardiovascular Dysfunction, and Steatosis.” American Journal of Physiology. Heart and Circulatory Physiology 310, no. 11: H1658–H1670. 10.1152/ajpheart.00214.2016. 27106042 PMC4935511

[fsn370798-bib-0032] McNulty, H. , M. Ward , L. Hoey , C. F. Hughes , and K. Pentieva . 2019. “Addressing Optimal Folate and Related B‐Vitamin Status Through the Lifecycle: Health Impacts and Challenges.” Proceedings of the Nutrition Society 78, no. 3: 449–462. 10.1017/S0029665119000661. 31155015

[fsn370798-bib-0033] Monserrat‐Mesquida, M. , M. Quetglas‐Llabrés , C. Bouzas , et al. 2022. “Effects of 2‐Year Nutritional and Lifestyle Intervention on Oxidative and Inflammatory Statuses in Individuals of 55 Years of Age and Over at High Cardiovascular Risk.” Antioxidants Basel, Switzerland 11, no. 7: 1326. 10.3390/antiox11071326. 35883817 PMC9312253

[fsn370798-bib-0034] Moustakli, E. , A. Zikopoulos , C. Skentou , et al. 2024. “Impact of Reductive Stress on Human Infertility: Underlying Mechanisms and Perspectives.” International Journal of Molecular Sciences 25, no. 21: 11802. 10.3390/ijms252111802. 39519353 PMC11547078

[fsn370798-bib-0035] National Cholesterol Education Program (NCEP) Expert Panel on Detection, Evaluation, and Treatment of High Blood Cholesterol in Adults (Adult Treatment Panel III) . 2002. “Third Report of the National Cholesterol Education Program (NCEP) Expert Panel on Detection, Evaluation, and Treatment of High Blood Cholesterol in Adults (Adult Treatment Panel III) Final Report.” Circulation 106, no. 25: 3143–3421. 12485966

[fsn370798-bib-0036] Nerurkar, P. V. , K. Gandhi , and J. J. Chen . 2021. “Correlations Between Coffee Consumption and Metabolic Phenotypes, Plasma Folate, and Vitamin B12: NHANES 2003 to 2006.” Nutrients 13, no. 4: 1348. 10.3390/nu13041348. 33919513 PMC8073624

[fsn370798-bib-0037] Niemann, B. , S. Rohrbach , M. R. Miller , D. E. Newby , V. Fuster , and J. C. Kovacic . 2017. “Oxidative Stress and Cardiovascular Risk: Obesity, Diabetes, Smoking, and Pollution: Part 3 of a 3‐Part Series.” Journal of the American College of Cardiology 70, no. 2: 230–251. 10.1016/j.jacc.2017.05.043. 28683970 PMC5568826

[fsn370798-bib-0038] Nordin, N. , Z. Zainol , M. H. Mohd Noor , and L. F. Chan . 2023. “An Explainable Predictive Model for Suicide Attempt Risk Using an Ensemble Learning and Shapley Additive Explanations (SHAP) Approach.” Asian Journal of Psychiatry 79: 103316. 10.1016/j.ajp.2022.103316. 36395702

[fsn370798-bib-0039] Ponce‐Bobadilla, A. V. , V. Schmitt , C. S. Maier , S. Mensing , and S. Stodtmann . 2024. “Practical Guide to SHAP Analysis: Explaining Supervised Machine Learning Model Predictions in Drug Development.” Clinical and Translational Science 17, no. 11: e70056. 10.1111/cts.70056. 39463176 PMC11513550

[fsn370798-bib-0040] Qi, X. , S. Wang , C. Fang , J. Jia , L. Lin , and T. Yuan . 2025. “Machine Learning and SHAP Value Interpretation for Predicting Comorbidity of Cardiovascular Disease and Cancer With Dietary Antioxidants.” Redox Biology 79: 103470. 10.1016/j.redox.2024.103470. 39700695 PMC11729017

[fsn370798-bib-0041] Rezk‐Hanna, M. , R. Gupta , C. O. Nettle , et al. 2022. “Differential Effects of Electronic Hookah Vaping and Traditional Combustible Hookah Smoking on Oxidation, Inflammation, and Arterial Stiffness.” Chest 161, no. 1: 208–218. 10.1016/j.chest.2021.07.027. 34298007 PMC8783031

[fsn370798-bib-0042] Rovin, B. H. , S. G. Adler , J. Barratt , et al. 2021. “KDIGO 2021 Clinical Practice Guideline for the Management of Glomerular Diseases.” Kidney International 100, no. 4, Supplement: S1–S276. 10.1016/j.kint.2021.05.021. 34556256

[fsn370798-bib-0043] Son, D.‐H. , H. S. Lee , S.‐Y. Seol , Y.‐J. Lee , and J.‐H. Lee . 2023. “Association Between the Oxidative Balance Score and Incident Chronic Kidney Disease in Adults.” Antioxidants (Basel, Switzerland) 12, no. 2: 335. 10.3390/antiox12020335. 36829895 PMC9952833

[fsn370798-bib-0044] Steven, S. , A. Daiber , J. F. Dopheide , T. Münzel , and C. Espinola‐Klein . 2017. “Peripheral Artery Disease, Redox Signaling, Oxidative Stress—Basic and Clinical Aspects.” Redox Biology 12: 787–797. 10.1016/j.redox.2017.04.017. 28437655 PMC5403804

[fsn370798-bib-0045] Surendran, A. , H. Zhang , T. Winter , A. Edel , H. Aukema , and A. Ravandi . 2019. “Oxylipin Profile of Human Low‐Density Lipoprotein Is Dependent on Its Extent of Oxidation.” Atherosclerosis 288: 101–111. 10.1016/j.atherosclerosis.2019.07.018. 31352271

[fsn370798-bib-0046] Talavera‐Rodriguez, I. , C. I. Fernandez‐Lazaro , Á. Hernández‐Ruiz , et al. 2023. “Association Between an Oxidative Balance Score and Mortality: A Prospective Analysis in the SUN Cohort.” European Journal of Nutrition 62, no. 4: 1667–1680. 10.1007/s00394-023-03099-8. 36781422 PMC10195723

[fsn370798-bib-0047] Terao, J. 2023. “Revisiting Carotenoids as Dietary Antioxidants for Human Health and Disease Prevention.” Food & Function 14, no. 17: 7799–7824. 10.1039/d3fo02330c. 37593767

[fsn370798-bib-0048] Uebanso, T. , T. Shimohata , K. Mawatari , and A. Takahashi . 2020. “Functional Roles of B‐Vitamins in the Gut and Gut Microbiome.” Molecular Nutrition & Food Research 64, no. 18: e2000426. 10.1002/mnfr.202000426. 32761878

[fsn370798-bib-0049] Wang, M. , and H. Shi . 2025. “Oxidative Balance Score Is Independently Associated With Reduced Prevalence of Sarcopenia Among US Adults With Metabolic Syndrome.” Frontiers in Nutrition 12: 1529140. 10.3389/fnut.2025.1529140. 40264554 PMC12011616

[fsn370798-bib-0050] Weinberg Sibony, R. , O. Segev , S. Dor , and I. Raz . 2024. “Overview of Oxidative Stress and Inflammation in Diabetes.” Journal of Diabetes 16, no. 10: e70014. 10.1111/1753-0407.70014. 39435991 PMC11494684

[fsn370798-bib-0051] Woolf, E. K. , J. D. Terwoord , N. S. Litwin , et al. 2023. “Daily Blueberry Consumption for 12 Weeks Improves Endothelial Function in Postmenopausal Women With Above‐Normal Blood Pressure Through Reductions in Oxidative Stress: A Randomized Controlled Trial.” Food & Function 14, no. 6: 2621–2641. 10.1039/d3fo00157a. 36847333

[fsn370798-bib-0052] Wronka, M. , J. Krzemińska , E. Młynarska , J. Rysz , and B. Franczyk . 2022. “The Influence of Lifestyle and Treatment on Oxidative Stress and Inflammation in Diabetes.” International Journal of Molecular Sciences 23, no. 24: 15743. 10.3390/ijms232415743. 36555387 PMC9778895

[fsn370798-bib-0053] Xu, X. , W. Wei , W. Jiang , et al. 2022. “Association of Folate Intake With Cardiovascular‐Disease Mortality and All‐Cause Mortality Among People at High Risk of Cardiovascular‐Disease.” Clinical Nutrition (Edinburgh, Scotland) 41, no. 1: 246–254. 10.1016/j.clnu.2021.11.007. 34929527

[fsn370798-bib-0054] You, Y. , Z. Wang , Z. Yin , et al. 2023. “Global Disease Burden and Its Attributable Risk Factors of Peripheral Arterial Disease.” Scientific Reports 13, no. 1: 19898. 10.1038/s41598-023-47028-5. 37963985 PMC10645774

[fsn370798-bib-0055] Zhang, W. , S.‐F. Peng , L. Chen , H.‐M. Chen , X.‐E. Cheng , and Y.‐H. Tang . 2022. “Association Between the Oxidative Balance Score and Telomere Length From the National Health and Nutrition Examination Survey 1999‐2002.” Oxidative Medicine and Cellular Longevity 2022: 1345071. 10.1155/2022/1345071. 35186180 PMC8850082

[fsn370798-bib-0056] Zhao, C. , L. Zhao , Y. Liu , et al. 2025. “The Impact of Serum Uric Acid on Biological Aging and Mortality Risk: Insights From the NHANES and CHARLS Cohorts.” Frontiers in Nutrition 12: 1569798. 10.3389/fnut.2025.1569798. 40331095 PMC12052535

[fsn370798-bib-0057] Zhou, R.‐H. , A. E. Vendrov , I. Tchivilev , et al. 2012. “Mitochondrial Oxidative Stress in Aortic Stiffening With Age: The Role of Smooth Muscle Cell Function.” Arteriosclerosis, Thrombosis, and Vascular Biology 32, no. 3: 745–755. 10.1161/ATVBAHA.111.243121. 22199367 PMC3288772

