# Supplementary material for: Evaluating the Oxidative Balance Score for Peripheral Artery Disease Risk: Integrating Epidemiologic Modeling and SHAP‐Interpretable Machine Learning in NHANES
Source: Food Sci Nutr. 2025 Aug 17;13(8):e70798. doi: 10.1002/fsn3.70798 (PMC12358680; doi:10.1002/fsn3.70798)
Supplement: Supplementary file 1 — Data S1: fsn370798‐sup‐0001‐Supinfo.pdf. [file FSN3-13-e70798-s001.pdf]

Supplementary Table S1. Association between total Oxidative Balance Score (OBS) and peripheral artery disease in the diabetic subgroup.

Groups	Crude model		Model 1		Model 2	
	HR (95% CI)	*P* value	HR (95% CI)	*P* value	HR (95% CI)	*P* value
Continuous	0.960(0.931,0.991)	0.013	0.966(0.931,1.003)	0.069	0.973(0.935,1.013)	0.180
Q1	ref		ref		ref	
Q2	0.463(0.265,0.806)	0.008	0.469(0.259,0.848)	0.014	0.464(0.247,0.873)	0.019
Q3	0.495(0.271,0.903)	0.023	0.571(0.285,1.142)	0.109	0.605(0.311,1.175)	0.132
Q4	0.583(0.319,1.068)	0.079	0.697(0.347,1.401)	0.301	0.738(0.334,1.631)	0.440
P for trend		0.054		0.254		0.376

Crude model: unadjusted for none. Model 1 adjusted for: ethnicity, age, sex, education, marital status. Model 2 adjusted for: ethnicity, age, sex, education, marital status, hypertension, hyperlipidemia, atherosclerotic cardiovascular disease, chronic kidney disease.

Supplementary Table S2. Association of dietary OBS and lifestyle OBS with PAD risk among diabetic participants.

Groups	Crude model		Model 1		Model 2	
	HR (95% CI)	*P* value	HR (95% CI)	*P* value	HR (95% CI)	*P* value
Lifestyle						
Continuous	0.919(0.794,1.064)	0.250	0.813(0.669,0.989)	0.039	0.797(0.638,0.994)	0.045
Q1	ref		ref		ref	
Q2	0.889(0.521,1.518)	0.659	0.721(0.372,1.397)	0.322	0.753(0.380,1.494)	0.404
Q3	1.171(0.678,2.021)	0.563	0.686(0.414,1.137)	0.139	0.736(0.424,1.275)	0.263
Q4	0.331(0.138,0.794)	0.015	0.228(0.079,0.661)	0.008	0.209(0.065,0.668)	0.010
P for trend		0.057		0.003		0.006
Dietary						
Continuous	0.960(0.928,0.994)	0.022	0.973(0.938,1.010)	0.145	0.981(0.942,1.022)	0.353
Q1	ref		ref		ref	
Q2	0.462(0.251,0.850)	0.014	0.520(0.262,1.029)	0.060	0.508(0.244,1.061)	0.070
Q3	0.425(0.224,0.807)	0.010	0.439(0.207,0.931)	0.033	0.480(0.219,1.049)	0.065
Q4	0.546(0.329,0.909)	0.021	0.760(0.432,1.338)	0.331	0.850(0.445,1.625)	0.613
P for trend		0.027		0.238		0.465

Crude model: unadjusted for none. Model 1 adjusted for: ethnicity, age, sex, education, marital status. Model 2 adjusted for: ethnicity, age, sex, education, marital status, hypertension, hyperlipidemia, atherosclerotic cardiovascular disease, chronic kidney disease.

Supplementary Figure S1. Restricted cubic spline analysis of total OBS and PAD risk among diabetic individuals.

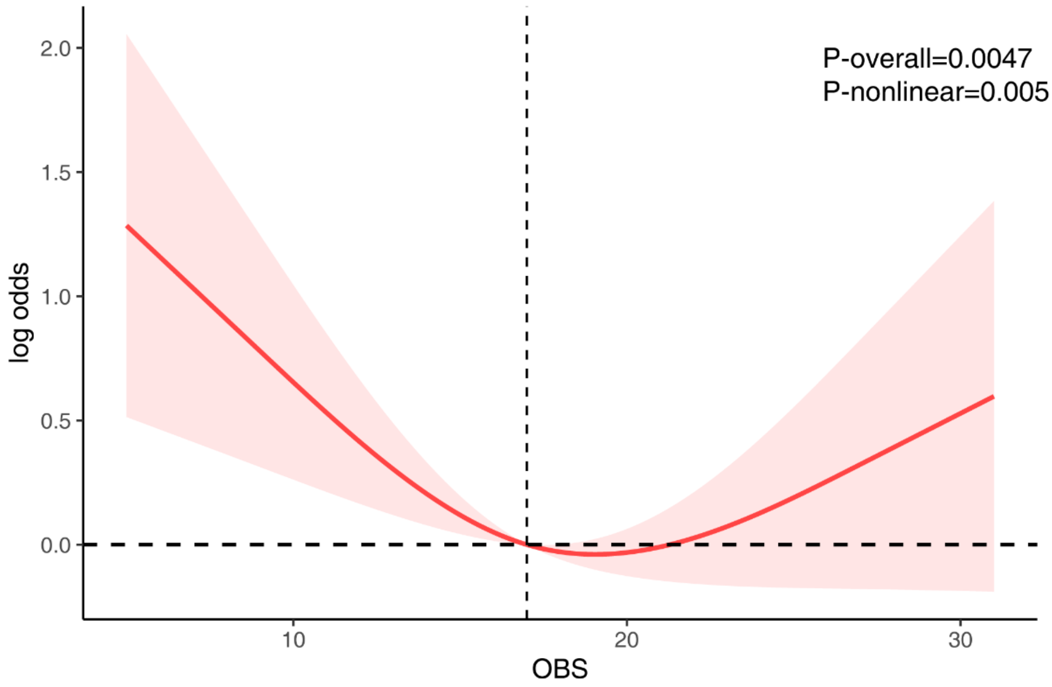

The model was adjusted for ethnicity, age, sex, education, marital status, hypertension, hyperlipidemia, atherosclerotic cardiovascular disease, and chronic kidney disease.

Supplementary Figure S2. Restricted cubic spline analysis of lifestyle OBS and PAD risk in the diabetic subgroup.

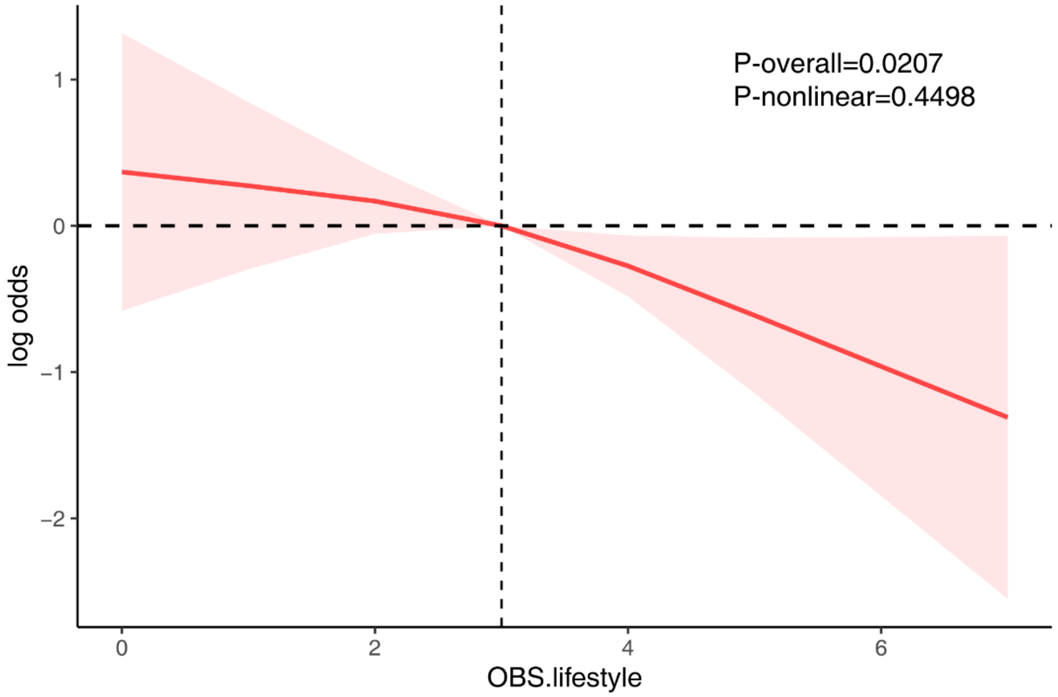

The model was adjusted for ethnicity, age, sex, education, marital status, hypertension, hyperlipidemia, atherosclerotic cardiovascular disease, and chronic kidney disease.

Supplementary Figure S3. Restricted cubic spline analysis of dietary OBS and PAD risk in the diabetic subgroup.

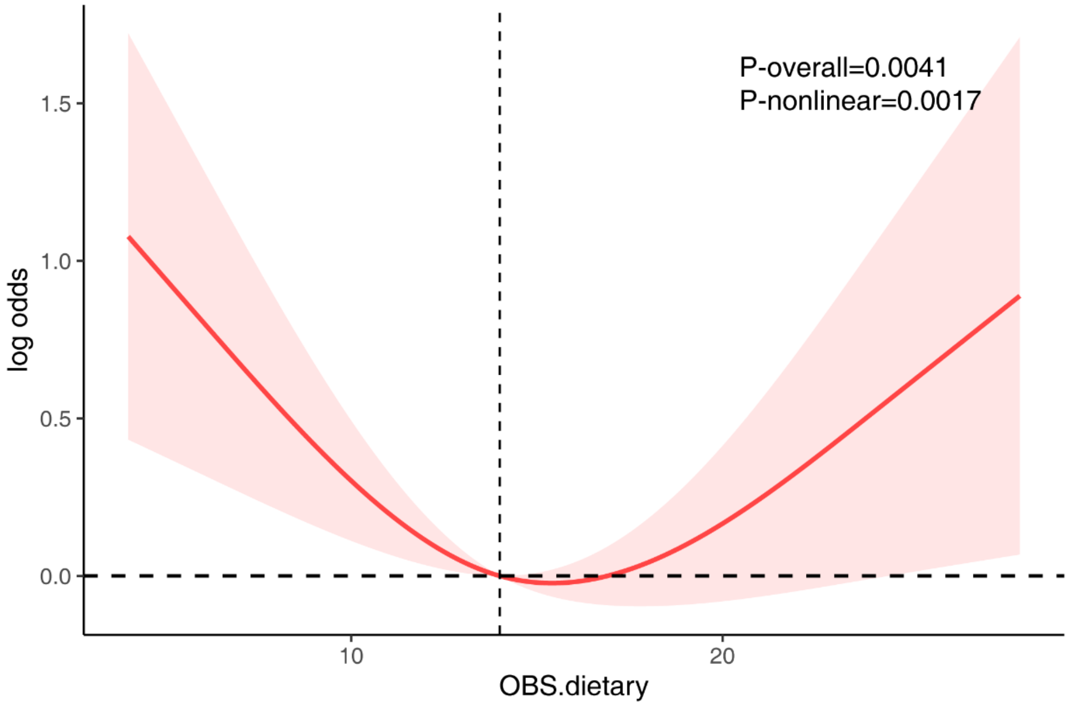

The model was adjusted for ethnicity, age, sex, education, marital status, hypertension, hyperlipidemia, atherosclerotic cardiovascular disease, and chronic kidney disease.
